# Brain xanthophyll content and exploratory gene expression analysis: subspecies differences in rhesus macaque

**DOI:** 10.1186/s12263-017-0557-3

**Published:** 2017-03-08

**Authors:** Emily S. Mohn, John W. Erdman, Martha Neuringer, Matthew J. Kuchan, Elizabeth J. Johnson

**Affiliations:** 1 Jean Mayer United States Department of Agriculture Human Nutrition Research Center on Aging, Tufts University, Boston, MA USA; 2 Department of Food Science and Human Nutrition, University of Illinois at Urbana-Champaign, Urbana, IL USA; 3 Division of Neuroscience, Oregon National Primate Research Center, Oregon Health and Science University, Beaverton, Oregon USA; 4 Abbott Nutrition, Columbus, Ohio USA; 5 Antioxidants Research Laboratory, 711 Washington Street, Boston, MA 02111 USA

**Keywords:** Lutein, Zeaxanthin, Rhesus monkey, Subspecies, Brain, RNA-sequencing

## Abstract

**Background:**

The dietary xanthophylls, lutein and zeaxanthin, accumulate in primate retina and brain, and emerging evidence indicates neural lutein content may be beneficial for cognition. Neural xanthophyll content in primates varies greatly among individuals, and genetic factors are likely to be significant contributors. Subspecies of rhesus macaques originating from different geographic locations are known to differ genetically, but the effect of origin on gene expression and carotenoid status has not been determined. The study objective was to determine whether xanthophyll status and expression of carotenoid-related genes, as well as genes with known variants between subspecies, differ between the brains of adult rhesus monkeys of Indian and Chinese origin.

**Methods:**

Samples of prefrontal cortex, cerebellum, and striatum were collected from adult monkeys (*n* = 9) fed a standard stock diet containing carotenoids. Serum and brain carotenoids were determined using reverse-phase high-performance liquid chromatography. For each brain region, RNA sequencing and real-time quantitative polymerase chain reaction were used to determine differentially expressed genes between the subspecies.

**Results:**

Indian-origin monkeys had higher xanthophyll levels in brain tissue compared to Chinese-origin monkeys despite consuming similar amounts of dietary carotenoids. In a region-specific manner, four genes related to carotenoid and fatty acid metabolism (*BCO2*, *RPE65*, *ELOVL4*, *FADS2*) and four genes involved in the immune response (*CD4*, *CD74*, *CXCL12 LTBR*) were differentially expressed between Indian- and Chinese-origin monkeys. Expression of all four genes involved in carotenoid and fatty acid metabolism were correlated with brain xanthophyll concentration in a region-specific manner.

**Conclusions:**

These results indicate that origin is related to differences in both gene expression and xanthophyll content in the brain. Findings from this study may have important implications regarding genetic diversity, lutein status, and cognition in primates.

**Electronic supplementary material:**

The online version of this article (doi:10.1186/s12263-017-0557-3) contains supplementary material, which is available to authorized users.

## Background

Lutein is a xanthophyll carotenoid found in a variety of colorful fruits and vegetables, as well as in dark leafy greens and eggs [1]. Animals cannot synthesize lutein, and therefore its presence in the body is a result of dietary consumption [2]. Lutein and its isomer zeaxanthin accumulate in the macula of the primate retina to form macular pigment (MP). In the macula, lutein and zeaxanthin function to protect the eye from oxidative damage both by filtering harmful blue light and by their antioxidant activity [3, 4]. More recently it has been discovered that lutein, and in smaller amounts, zeaxanthin, also preferentially accumulate in the primate brain [5–7]. Current evidence indicates that lutein may have an important role in maintaining and improving brain function and cognition, with studies showing that MP optical density (MPOD) [8–11], as well as serum and brain lutein concentrations, are associated with better cognition in humans [6].

It is well established that there is a high amount of inter-individual variability in serum carotenoid levels [12, 13]. A major contributing factor is the large variability in intestinal absorption of carotenoids [14]. Additionally, MP response to dietary and supplemental lutein/zeaxanthin is highly variable [15–17]. Genomic studies have identified a number of single nucleotide polymorphisms (SNPs) in genes involved in carotenoid uptake, transport, and metabolism that are related to MPOD and risk for age-related macular degeneration (AMD) [18, 19]. While a number of studies have documented significant variability in efficiency of uptake and storage of lutein and zeaxanthin in the eye, it is not clear whether the same variability is observed in the brain. However, it is likely that these tissues share similar mechanisms for xanthophyll uptake and deposition given that lutein is selectively taken up into this tissue just as it is in the retina [6, 7]. Evidence to support this hypothesis has been shown in both human and non-human primates where MP was significantly correlated with lutein concentrations in the brain [20, 21].

Like humans, but unlike other animal models, rhesus macaques (*Macaca mulatta*) have been shown to absorb and preferentially store xanthophylls in the eye and brain [20, 22], making them an ideal model for studying factors influencing the accumulation of lutein and zeaxanthin into neural tissue. Another advantage of using these non-human primates is that rhesus monkeys of different geographical origin (Indian and Chinese) have been shown to be genetically different, with a number of SNPs distinguishing the two subspecies from one another [23]. Therefore, utilizing both subspecies of monkeys in carotenoid research provides a unique opportunity for studying the nutrigenomics of xanthophyll absorption, deposition, and storage/metabolism in neural tissue. However, to date, no studies have investigated whether accumulation of xanthophylls in neural tissue differs between rhesus monkeys of Indian and Chinese origin when provided very similar carotenoid-containing foods. Additionally, no studies have determined the relationship between rhesus monkey origin and expression of genes related to xanthophyll metabolism and function.

The objective of this study was twofold: (1) to determine whether xanthophyll status in serum and brain differs between rhesus monkeys of different subspecies and (2) to investigate whether the brains of rhesus monkeys of Indian and Chinese origin differs in the expression of genes involved in carotenoid uptake, transport, and metabolism as well as genes with known variants between subspecies. Brain regions of interest included the prefrontal cortex (PFC), cerebellum (CER), and striatum (STR), since these regions play important but distinct roles in cognitive function [24–26].

## Methods

### Study animals and diet

Post-mortem brain samples from nine rhesus monkeys (*M. mulatta*) ranging from 7 to 16 years of age were obtained from the Tissue Distribution Program at the Oregon National Primate Research Center (ONPRC) at Oregon Health and Science University. All animals were fed a standard monkey stock diet (LabDiet #5037, St. Louis, MO) that was determined by reverse-phase high-performance liquid chromatography (HPLC) [27] to contain ~16 μmol/kg of lutein, ~6 μmol/kg of zeaxanthin, ~5 μmol/kg β-carotene, ~1 μmol/kg α-carotene, and ~0.1 μmol/kg cryptoxanthin, plus a variety of supplemental fruits and vegetables two times/week (seasonal: bananas, apples, oranges, spinach, cucumber, carrots). All Indian-origin monkeys were born at the ONPRC, while monkeys of Chinese origin were obtained as young adults and lived for 3 years at the ONPRC prior to termination. After termination, PFC, CER, and STR were dissected, frozen at −0 °C and shipped on dry ice overnight to Tufts University for analysis. Tissues were kept frozen at −80 °C until analysis.

### Carotenoid extraction from brain regions and serum

Extraction of carotenoids from brain regions was adapted from previous methods [28] and has been described in detail [20]. Seven of the nine monkeys had serum collected at termination that was available for carotenoid analysis. Carotenoids were extracted from serum as previously described [29]. HPLC was used to separate and quantify carotenoids [29]. For serum, total lutein (sum of *cis* and *trans* isomers) was used for the data analysis. In the brain, only the *trans* isomer of lutein was detected. For the other carotenoids, including zeaxanthin, only the *trans* isomers were detected in serum and brain. Carotenoid data (both serum and brain) are expressed as mean ± standard error of mean.

### Total RNA extraction from rhesus monkey brain for RNA sequencing and RT-PCR

From the nine monkeys, three from each subspecies were selected for RNA sequencing (RNA-seq) analysis that were matched for age (13 ± 4 years and 11 ± 1 year for Indian- and Chinese-origin monkeys, respectively) and sex (two females and one male in each group). Total RNA was isolated from PFC, CER, and STR using the RNeasy lipid tissue mini kit (Qiagen) per manufacturer’s instructions. RNA purity and concentrations were determined using a NanoDrop ND-1000 (Thermo Fisher Scientific). All RNA samples had a 260/280 ratio greater than 2.05. One to 2 μg RNA was aliquoted for RNA-seq analysis. RNA integrity was determined to be satisfactory for sequencing using an AATI (Advanced Analytical Technologies, Inc) Fragment Analyzer. One microgram RNA was aliquoted for real-time quantitative polymerase chain reaction (RT-PCR).

### Library preparation, next-generation RNA sequencing, and processing of reads

RNA-seq was performed in order to explore differences in gene expression profiles in the brains of rhesus monkeys of different origin. RNA-seq uses next-generation sequencing to characterize genome-wide RNA sequences and determine their abundance in a given sample with extremely high resolution. Due to its high sensitivity and broad scope, RNA-seq is quickly becoming the preferred method over microarrays for differential gene expression studies [30].

For library preparation, the TruSeq RNA library preparation kit (Illumina, Inc) was used according to the manufacturer’s protocol. Single-end 50-bp sequencing was performed on the HiSeq 2500, High Output v4 (eight lanes flow cell) system. For this analysis six samples were sequenced per lane (total of three lanes used, one for each brain region). Quality control of the resulting reads was performed using the FastQC tool on the Tufts Galaxy server. Additional file 1: Table S1 shows the average number of reads and the mean quality score (PHRED format) for Indian- and Chinese-origin groups for each brain region.

### Mapping of RNA-seq reads using TopHat

Sequence reads were aligned to the Ensembl rhesus monkey reference genome (mmul1) using TopHat for Illumina (version 1.5.0) on the Tufts University Galaxy server. TopHat aligns reads to mammalian-sized genomes using the high-throughput short read aligner Bowtie and then analyzes the mapping results to identify splice junctions between exons [31]. Default settings for TopHat were used.

### Differential expression testing using Cuffdiff

Using the resulting BAM files (.bam) containing mapped reads from TopHat and a reference *M. mulatta* iGenome GTF annotation file, transcripts were assembled and normalized to fragments per kilobase of exon per million fragments (FPKM) expression units to estimate the relative abundance of transcripts. Differential expression of FPKM estimates was determined using Cuffdiff [32]. Differential gene expression is expressed as fold change between Indian- and Chinese-origin monkeys. Differences in gene expression between monkeys of different origin were determined using two-tailed Student’s *t* tests. Resulting *p* values were adjusted for multiple testing using the Benjamini-Hochberg (false discovery rate) correction (*q* value).

### Validation using RT-PCR

Genes with statistically significant (*P* < 0.05) fold changes ranging from 1.25 to 3.69 (for upregulated genes) and −1.52 to −2.44 (for downregulated genes) between Indian and Chinese-origin monkeys were selected for differential expression analysis using real-time quantitative polymerase chain reaction. Complementary DNA (cDNA) was generated from total RNA (1 μg) and RT-PCR was performed using SYBR Green (Applied Biosystems 7300, Carlsbad, CA). Relative expression was calculated using the 2^–ΔΔCT^ method. After testing three different reference genes (actin gamma 1 (*ACTG1*), ribosomal protein L13a (*RPL13a*), and alpha-1,2-mannosyltransferase (*ALG9*)), *ACTG1* was determined to have the lowest variability between monkeys of different origins and among different brain regions. Therefore, it was chosen as the endogenous control for this study. Primer sequences are listed in Additional file 1: Table S2. The data is expressed as the relative RNA expression of Chinese- to Indian-origin monkeys.

### Statistical analysis

Two-tailed student’s *t* test was performed to determine differences in each carotenoid and in gene expression between monkeys of different origins. Pearson’s correlation analysis was performed to determine the association between brain xanthophyll concentration and gene expression. Given the exploratory nature of this pilot study, significance was set at the 0.1 level.

## Results

### Carotenoid profile in serum

**Fig. 1:**
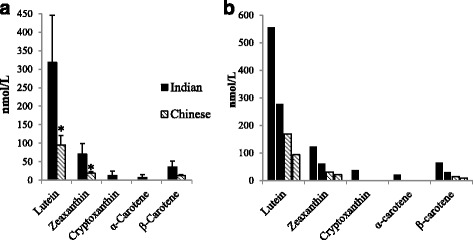
Mean (±SEM) carotenoid content in serum of rhesus macaque of different origin in **a** (*n* = 4 Chinese, *n* = 3 Indian) and **b** subset used for gene expression analysis (*n* = 2 for each origin). Lutein (cis+trans) and zeaxanthin (trans, no cis present) concentrations (nmol/L) were greater in monkeys of Indian origin vs. Chinese (Fig. 1a. *p < 0.09, Student’s *t* test)

Mean carotenoid profiles in serum of all Indian- and Chinese-origin monkeys for which samples were available (*n* = 7; three Indian-origin, four Chinese-origin) are shown in Fig. 1a. Lutein was the major carotenoid detected in serum for both Indian- (318 ± 127 nmol/L) and Chinese-origin (94.3 ± 26.5 nmol/L) rhesus monkeys. Other carotenoids detected in both subspecies included zeaxanthin (70.2 ± 28.7 nmol/L and 18.6 ± 5.0 nmol/L, Indian and Chinese, respectively) and β-carotene (35.7 ± 15.5 nmol/L and 11.90 ± 2.86 nmol/L, Indian and Chinese respectively). Cryptoxanthin and α-carotene were only detected in one monkey, which was of Indian origin. Between subspecies, lutein and zeaxanthin concentrations were greater in monkeys of Indian origin compared to Chinese origin (*p* = 0.09). β-carotene concentrations also tended to be higher in monkeys of Indian origin, but this was not statistically significant (*p* = 0.14).

Serum carotenoid profiles in the subset of monkeys selected for brain gene expression analysis are shown in Fig. 1b (*n* = 4, two Indian-origin, two Chinese-origin). The average lutein concentration in the Indian-origin monkeys was 417 nmol/L, over three times greater than the average concentration of the Chinese-origin monkeys (132 nmol/L). For zeaxanthin, the average concentration in Indian-origin monkeys was 92.6 nmol/L while the concentration in Chinese monkeys was 26.4 nmol/L. Serum β-carotene concentration was four times higher in Indian-(47.7 nmol/L) versus Chinese-origin monkeys (11.9 nmol/L).

### Lutein and zeaxanthin concentration in the brain

**Fig. 2:**
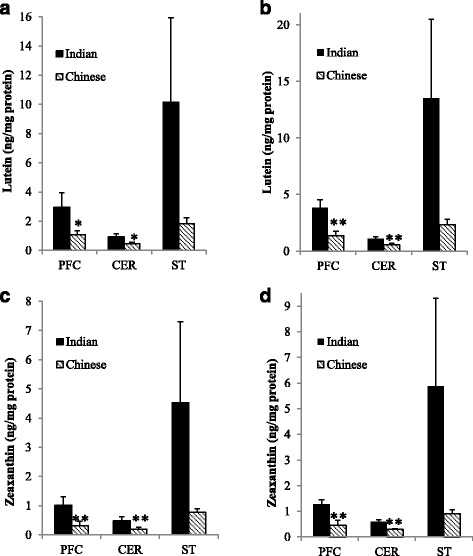
Mean xanthophyll concentrations (ng/mg protein) ±SEM in prefrontal cortex (PFC), cerebellum (CER), and striatum (STR) of rhesus macaques of different origin. Lutein concentrations were greater in monkeys of Indian origin compared to Chinese in PFC and CER in **a** all monkeys analyzed (*p < 0.1, Student’s *t* test, *n* = 5 Indian, *n* = 4 Chinese) and **b** in subset of monkeys selected for gene expression analysis (**p < 0.05, Student’s *t* test, *n* = 3 per group). Zeaxanthin concentrations were greater in monkeys of Indian origin compared to Chinese in PFC and CER in **c** all monkeys analyzed (*n* = 9) and **d** in subset of monkeys selected for gene expression analysis (*n* = 6), **P < 0.05 for both, Student’s *t* test

Lutein and zeaxanthin concentrations in PFC, CER, and STR of all Indian-(*n* = 4) and Chinese-origin (*n* = 5) monkeys are shown in Fig. 2a and c, respectively. Rhesus monkeys had the same distribution pattern of lutein and zeaxanthin among the three regions regardless of origin. Namely, xanthophyll levels tended to be the highest in STR, followed by PFC and the lowest in CER (Indian origin *p* = 0.1; Chinese origin *p* < 0.05). In PFC and CER, lutein concentrations were greater in monkeys of Indian origin compared to those of Chinese origin (Fig. 2a, *p* = 0.08 for both). Similarly, zeaxanthin concentrations were higher in the PFC and CER of these animals compared to Chinese (Fig. 2c, *p* < 0.05 for both). Lutein and zeaxanthin concentrations also tended to be higher in the STR of Indian-origin monkeys compared to Chinese origin; however, this was not statistically significant (*p* = 0.14 and *p* = 0.16, respectively).

Lutein and zeaxanthin concentrations in PFC, CER, and STR of Indian- and Chinese-origin monkeys selected for gene expression analysis (*n* = 3 per group) are shown in Fig. 2b and d, respectively. Both lutein and zeaxanthin concentrations were significantly higher in PFC and CER of Indian-origin monkeys compared to Chinese-origin (*p* < 0.05 for all comparisons). Individual xanthophyll concentrations for this subset of rhesus monkeys are shown in Additional file 2: Figures S1, S2, and S3.

### Differences in gene expression between monkeys of Indian and Chinese origin using RNA-Seq

Given that xanthophyll concentrations differ in the brain between Indian- and Chinese-origin rhesus monkeys, we elected to focus this analysis on genes that are known to be related to carotenoid uptake/transport (*NPC1L1*, *ABCG5*, *ABCA1*, *SCARB1*, *LIPC*), carotenoid binding in neural tissue (*GSTP1*, *STARD3*) and carotenoid metabolism (*BCO1*, *BCO2*), as well as genes involved in long-chain omega-3 polyunsaturated fatty acid (PUFA) metabolism and synthesis (*ELOVL2/4/5* and *FADS1/2*), which have been shown to be related to xanthophyll uptake and accumulation in neural tissue [18, 19] and genes related to maculopathies and low macular xanthophyll status (*RPE65*, *ALDH3A2*) (Table 1). In the PFC, *BCO2* (1.64 fold change, *p* < 0.01) and *ELOVL4* (1.25 fold change, *p* < 0.05) have higher expression in monkeys of Chinese origin compared to Indian origin. Similarly, in the striatum, *BCO2* expression was higher in Chinese-origin monkeys compared to Indian (1.69 fold change, *p* = 0.01). In this region, expression of the fatty acid desaturase genes *FADS1* (1.55 fold change, *p* = 0.01) and *FADS2* (1.32 fold change, *p* = 0.05), as well as the cholesterol transporter, *ABCA1* (1.43 fold change, *p* = 0.06), and receptor, *SCARB1* (1.20 fold change, *p* = 0.09), were also higher in Chinese monkeys compared to Indian. In the CER, only *RPE65* had a significant fold change, with lower expression (−2.44 fold change, *p* = 0.03) in monkeys of Chinese origin compared to Indian origin. However, all gene expression differences lost statistical significance when adjusted for multiple comparisons (*q* > 0.1).

**Table 1:** Relative expression of genes (fold change, Chinese vs. Indian) involved in xanthophyll uptake and metabolism in prefrontal cortex (PFC), cerebellum (CER), and striatum (STR) of Indian and Chinese-origin rhesus monkeys (*n* = 3 per group)

	Fold change-PFC	*p* value	Fold change-CER	*p* value	Fold change-STR	*p* value
BCO1	−1.15	0.77	2.75	0.99	1.84	0.37
**BCO2**	*1.64*	*0.006*	1.58	0.13	*1.69*	*0.01*
NPC1L1	1.54	0.34	−1.06	0.91	1.26	0.60
ABCG5	−1.37	0.99	1.17	0.68	1.06	0.91
*ABCA1*	1.05	0.84	−1.01	0.99	*1.43*	*0.06*
*SCARB1*	1.18	0.12	1.10	0.43	*1.20*	*0.09*
GSTP1	1.07	0.52	−1.09	0.59	1.15	0.38
STARD3	1.09	0.99	1.07	0.99	1.31	0.99
LIPC	−1.61	0.25	1.38	0.99	−1.37	0.46
ELOVL2	−1.02	0.87	1.06	0.71	1.08	0.67
**ELOVL4**	*1.25*	*0.04*	1.06	0.66	1.11	0.50
ELOVL5	1.14	0.49	−1.05	0.81	1.24	0.29
**FADS1**	1.17	0.15	1.20	0.20	*1.55*	*0.01*
**FADS2**	1.16	0.12	−1.04	0.75	*1.32*	*0.05*
**RPE65**	−1.28	0.44	*−2.44*	*0.03*	−1.37	0.26
ALDH3A2	−1.11	0.26	−1.05	0.61	1.02	0.89

Fold change calculated by dividing fragments per kilobase of transcript per million mapped reads (FPKM) Chinese origin by FPKM Indian origin. Genes in italics showed a significant difference in expression in at least one brain region. Genes in bold were selected for RT-PCR analysis

A number of known single nucleotide polymorphisms (SNPs) have previously been identified between rhesus monkeys of Indian and Chinese origin [23]. However, whether there are also differences in expression of these genes between the different subspecies has not yet been determined. Therefore, we also elected to look at differences in expression of these genes (Table 2). Of the 27 genes analyzed, 9 were differentially expressed in at least one brain region. In all three regions, *CD74* and *CD4*, genes that encode proteins important for antigen presentation and immune function, were differentially expressed (*p* ≤ 0.0001), with expression levels higher in Chinese monkeys compared to Indian. In STR, *CXCL12*, a gene encoding a chemokine, expression was also significantly higher in Chinese-origin monkeys (3.22 fold change, *p* < 0.001). Fold change expression of these three genes in PFC and STR remained statistically significant after adjusting for multiple comparisons (*q* < 0.01).

**Table 2:** Relative expression of genes (fold change, Chinese vs. Indian) with known variants between rhesus monkeys of different origin in the prefrontal cortex (PFC), cerebellum (CER), and striatum (STR) (*n* = 3 per group)

	Fold change-PFC	*p* value	Fold change-CER	*p* value	Fold change-STR	*p* value
CCL5	1.07	0.98	1.01	0.99	1.15	0.94
**CXCL12**	1.45	0.19	1.18	0.61	*3.22*	*0.00058*
XCL1	1.43	0.42	0.89	0.77	3.57	0.11
CCR4	1.50	0.67	1.27	0.72	1.19	0.79
**CCR1**	*1.94*	*0.02*	1.46	0.21	*1.57*	*0.07*
IL2RA	2.04	0.50	1.89	0.58	1.00	0.98
**CD74**	*2.11*	*0.0001*	*1.44*	*0.09*	*2.52*	*0.0000043*
**CD4**	*3.69*	*0.0000001*	*1.66*	*0.008*	*3.59*	*0.0000006*
CD44	−1.08	0.81	−1.12	0.31	1.27	0.38
TLR5	1.06	0.83	−1.20	0.63	1.02	0.94
**LTBR**	*−1.52*	*0.02*	−1.28	0.22	−1.06	0.80
FAS	1.27	0.58	1.24	0.57	−1.39	0.43
MAOA	1.06	0.72	1.03	0.89	1.04	0.86
BCHE	1.13	0.15	1.43	0.12	−1.01	0.99
*NOS1*	1.06	0.82	1.24	0.25	*1.57*	*0.05*
*NPY*	1.00	0.99	*−10.0*	*0.05*	1.18	0.36
PYY	1.08	0.92	1.92	0.65	3.04	0.41
SLC5A7	1.56	0.19	1.67	0.19	1.01	0.95
SLC6A4	1.67	0.67	−3.23	0.27	−1.75	0.69
**SNCA**	*1.21*	*0.10*	1.08	0.63	*1.41*	*0.04*
INHBB	−1.11	0.68	−1.08	0.82	1.13	0.68
SIRT1	1.13	0.51	1.08	0.64	1.23	0.34
HTATSF1	1.10	0.35	1.03	0.82	1.13	0.41
STAR	−1.52	0.18	−1.30	0.49	−1.04	0.91
*ADRBK2*	1.21	0.19	1.11	0.49	*1.52*	*0.08*
ITGA4	−1.10	0.74	1.01	0.99	1.80	0.32
SASH1	1.13	0.35	1.21	0.31	1.30	0.12

Fold change calculated by dividing fragments per kilobase of transcript per million mapped reads (FPKM) Chinese origin by FPKM Indian origin. Genes in italics showed a significant difference in expression in at last one brain region. Genes bold were selected for RT-PCR analysis

Expression of *CCR1*, gene encoding a chemokine receptor, was also greater in the PFC (1.94 fold change, *p* = 0.02) and STR (1.57 fold change, *p* = 0.07) of Chinese-origin monkeys compared to Indian-origin animals. Another gene related to inflammation, and a member of the tumor necrosis factor (TNF) family, *LTBR*, was differentially expressed in PFC, with expression being lower in monkeys of Chinese origin. However, differences in expression of both these genes lost significance after adjusting for multiple comparisons (*q* > 0.1). Three genes related to neurotransmission (*SNCA*, *NOS1*, *NPY*) were differentially expressed. Specifically, *SNCA*, which encodes a protein (alpha-synuclein) that regulates presynaptic transmission, was observed to have higher expression in monkeys of Chinese origin in both PFC (1.21 fold change, *p* = 0.1) and STR (1.41 fold change, *p* = 0.04). *NOS1* expression was higher in Chinese-origin monkeys compared to Indian (1.57 fold change, *p* = 0.05) while *NPY* expression was significantly lower in Chinese monkeys (−10.0 fold change, *p* = 0.05). Again, differences in expression lost significance after adjusting for multiple comparisons (*q* > 0.1).

### Differences in gene expression between monkeys of Indian and Chinese origin using RT-PCR

Although no carotenoid-related genes had significant *q* values between monkeys of different origin, we elected to perform differential gene expression analysis using RT-PCR for genes with expression difference *p* values ≤0.05 (*BCO2*, *ELOVL4*, *FADS1*, *FADS2*, *RPE65*). Similarly, for genes with known variants between rhesus monkeys of Indian and Chinese origin, we performed differential gene expression analysis not only on genes with a significant *q* value (*CXCL12*, *CD74*, *CD4*), but also genes with expression differences *p* < 0.05 (*LTBR*, *CCR1*, *SNCA*).

**Fig. 3:**
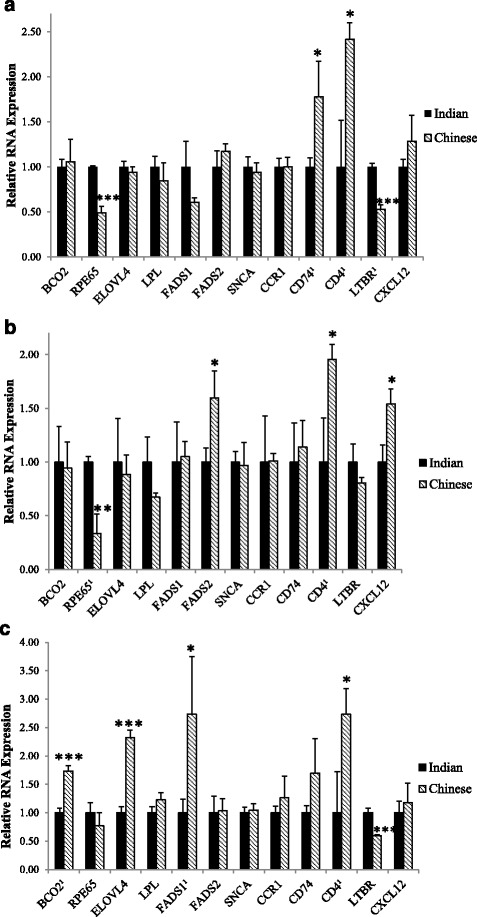
Relative Expression (±SEM) of genes selected for RT-PCR analysis in. **a** Prefrontal Cortex. **b** Cerebellum. **c** Striatum of Indian and Chinese-origin rhesus monkeys (*n* = 3 per origin). ***p < 0.01, **p < 0.05, *p < 0.1. ^*1*^ *Superscript* next to gene name indicates significant *p* value also observed in RNA-seq analysis for the same brain region

Results from RT-PCR analysis are shown in Fig. 3a–c. In the PFC, *RPE65* expression was significantly lower in monkeys of Chinese origin compared to those of Indian origin (*p* < 0.01). Regarding genes with known variants between subspecies, *CD74*, *CD4*, and *LTBR* were differentially expressed. Specifically, *CD74* and *CD4* expression was higher in Chinese-origin monkeys compared to Indian origin (*p* < 0.1). Conversely, *LTBR* expression was significantly lower in these monkeys compared to those of Indian origin (*p* < 0.01). In the CER, expression of *RPE65* was also lower in Chinese-origin monkeys compared to Indian (*p* < 0.05). Additionally, *FADS2* expression was higher in Chinese-origin monkeys (*p* < 0.1). Higher expression of *CD4* (*p* < 0.1) and *CXCL12* (*p* = 0.06) was also observed in monkeys of Chinese origin. In the STR, 5 of the 12 genes were differentially expressed. Differentially expressed genes related to carotenoid uptake, transport, and metabolism included *BCO2* (*p* < 0.01), *ELOVL4* (*p* < 0.01), and *FADS1* (*p* < 0.1), all of which had higher expression in monkeys of Chinese origin. Differential expression of genes with known variants between Indian and Chinese origin included *CD4* (*p* < 0.1) and *LTBR* (*p* < 0.01), the former of which had higher expression and the latter, lower expression, in monkeys of Chinese origin compared to Indian origin. In Fig. 3, superscripts next to gene names indicate consistency in results between RNA-seq and RT-PCR analysis within each brain region.

In order to examine the cross-sectional relationship between brain xanthophyll content and expression of genes involved in carotenoid and fatty acid metabolism, correlation analyses was performed for each brain region (Table 3). Results from this analysis indicate positive associations between xanthophyll concentration and *RPE65* expression in PFC and CER (*p* < 0.05). In STR, xanthophyll concentration was inversely associated with *BCO2* expression (*p* < 0.05) and *ELOVL4* (*p* < 0.1). Additionally, *FADS1* and *FADS2* were negatively associated with xanthophyll content in STR and CER, respectively (*p* < 0.1).

**Table 3:** Cross-sectional relationship between xanthophyll concentration (ng/mg protein) and gene expression (fold change) in rhesus monkeys of different origin in prefrontal cortex (PFC), cerebellum (CER), and striatum (STR) (*n* = 6)

	BCO2	RPE65	ELOVL4	FADS1	FADS2
Prefrontal cortex	–	0.84**	0.65	0.58	−0.67
Cerebellum	–	0.83**	–	–	−0.68*
Striatum	−0.86**	–	−0.68*	−0.77*	–

*P ≤ 0.1

**P < 0.05

## Discussion

### Xanthophyll status differs between rhesus monkeys of different origin

Indian-origin monkeys were found to have higher xanthophyll content in serum, with a combined 3.5 times greater concentration of xanthophylls, compared to monkeys of Chinese origin, despite eating the same stock diet. Although we cannot rule out a contribution of modest differences in preference for seasonal fruits and vegetables, it is much more likely that these differences are due to differing efficiencies in their ability to absorb, transport, and metabolize lutein and zeaxanthin.

Indian-origin monkeys were also observed to have higher xanthophyll concentrations in each brain region compared to Chinese-origin monkeys. However, variability within each group was high, particularly for the ST of Indian-origin monkeys. Therefore, origin does not account for all of the variability observed with neural lutein content among primates. In addition, monkeys of both subspecies tended to have lower levels of lutein in CER compared with PFC and ST, two regions more closely linked to cognitive function. Specifically, lutein is known to be related to improved working memory, executive function, and language in humans, all of which involve function of the PFC and STR [6, 33].

### Expression of carotenoid-related genes in rhesus monkey brain

As determined by PCR, differential gene expression analysis indicated region-specific differences in expression of four carotenoid metabolism genes in Chinese- versus Indian-origin monkeys. The CER had the fewest number of differentially expressed carotenoid-related genes, only *RPE65* and *FADS2*, which is consistent with this region having the smallest difference in xanthophyll concentrations (~2 times higher in Indian vs. Chinese) compared to the other regions (~3 times in PFC, ~5.5 in STR), which had a combined four differentially expressed genes (*BCO2*, *RPE65*, *ELOVL4*, *FADS1*). Our findings also indicate that brain xanthophyll concentration is directly associated with expression of these genes in a region-specific manner.

The *BCO2* gene encodes a mitochondrial enzyme responsible for the degradation of carotenoids. Evidence indicates that this enzyme is induced to prevent over-accumulation of carotenoids in mitochondria, which can lead to mitochondrial dysfunction and oxidative stress [34]. Higher expression of this gene in animals of Chinese origin versus Indian origin and an inverse correlation between xanthophyll concentration and *BCO2* expression in STR suggest that differences in neural xanthophyll content between origins may be due, in part, to differences in metabolism/degradation. The *RPE65* gene also encodes a carotenoid oxygenase, which has a well-established function in the retina as a critical participant in the visual cycle where it produces 11-*cis*-retinol [35]. This gene has been linked to macular pigment response to supplementation, and thus, it appears to be related to retinal lutein and zeaxanthin content in some capacity [17, 18]. Mutations in this gene are linked with blindness and retinal degenerations, including Leber congenital amaurosis and retininitis pigmentosa [36]. Our findings indicate that *RPE65* expression is also related to lutein and zeaxanthin content in the brain. However, it is not clear what function this enzyme possesses in brain tissue. Therefore, it is difficult to speculate on the relationship between brain xanthophyll content and *RPE65* expression at this time.

*FADS1* and *FADS2* encode the enzymes delta-5 desaturase and delta-6 desaturase, respectively. They are the rate-limiting enzymes and main determinants for the synthesis of long-chain polyunsaturated fatty acids (PUFA), including eicosapentaenoic acid (EPA) and docosahexaenoic acid (DHA), from their precursors [37]. *ELOVL4* mediates fatty acid elongation, in particular, the synthesis of very-long-chain PUFAs (carbon chain length 24–36), which are present in neural retina membranes. Circulating levels of EPA and docosapentaenoic acid (DPA) [38] and SNPs in all three genes are associated with MPOD [18]. Furthermore, *ELOVL4* mutations underlie autosomal-dominant Stargardt disease, a form of early-onset macular degeneration [39]. Given that lutein and zeaxanthin are likely taken up into the brain through similar mechanisms as in the eye, there may be a similar relationship in both tissues between long-chain PUFAs (particularly EPA and DHA) and xanthophyll concentrations (or vice versa). This evidence is also consistent with previous findings that brain concentrations of lutein and DHA may interact with one another to influence cognitive function in humans [40, 41]. One potential explanation for the inverse associations between xanthophyll concentration and expression of *ELOVL4*, *FADS1*, and *FADS2* may be a compensatory mechanism. That is, lower xanthophyll levels in the brain may trigger upregulation of enzyme expression to maintain functions common to xanthophylls and long-chain PUFAs, such as membrane fluidity or anti-inflammatory actions. However, the mechanisms underlying the relationship between brain lutein and PUFA composition remain unclear, and cause-and-effect relationships cannot be determined from these data.

### Brain expression of genes with SNPs between Indian- and Chinese-origin rhesus monkeys

Given the well-established genetic divergence between Indian- and Chinese-origin monkeys, we sought to determine whether genes with known SNPs between subspecies also differed in their expression levels in brain tissue. Our findings indicate that among the 27 identified genes with SNPs, three genes related to immune function (*CD4*, *CD74*, *LTBR*) and one related to chemokine expression (*CXCL12*) were differentially expressed between Indian- and Chinese-origin monkeys in a brain region-specific manner. *CD4* expression showed the most consistent expression differences, differing significantly in all three brain regions as evaluated by both RNA-seq and PCR. Higher expression of *CD4*, *CD74*, *CXCL12*, and lower expression of *LTBR* in Chinese-origin monkeys is consistent with what is known regarding simian immune-deficiency virus (SIV) infection and progression susceptibility in the two subspecies. Specifically, Chinese-origin monkeys are more resistant to SIV with slower disease progression than their Indian-origin counterparts [42–45]. Our findings support evidence that Chinese-origin monkeys may have stronger immune responses, particularly in the face of SIV infection.

Studies in diverse animal models (mice, dogs, cats, and birds) show enhanced immune responses with lutein supplementation [46]. With regard to HIV specifically, limited data indicates that circulating concentrations of lutein and zeaxanthin are lower in individuals with HIV versus controls [47]. However, all of these studies focused on systemic immune function, with no current evidence to date on the relationship between lutein and immunity in the brain. Research on age-related macular degeneration (AMD) in humans has provided evidence that the alternative complement pathway underlies the pathogenesis of AMD, and this pathway can be suppressed by the xanthophylls [46, 48]. Given that Chinese-origin monkeys may have more efficient immune systems than monkeys of Indian origin, it is possible they require, and therefore accumulate, less lutein and zeaxanthin for normal function in brain tissue.

## Conclusions

To our knowledge, this is the first study to explore the effect of genetic divergence (i.e., monkey subspecies based on geographic origin) on lutein concentrations and gene expression profiles in the primate brain. Findings from this study indicate that rhesus monkeys of Indian and Chinese origin differ in serum and brain lutein and zeaxanthin status despite eating the same standard stock diet during their adult life. Furthermore, neural xanthophyll content may be related to expression of genes involved in carotenoid and fatty acid metabolism in the brain. However, given that this is an exploratory pilot study, these results must be replicated with a larger sample size to confirm this relationship. Future studies determining whether these differences in gene expression actually translate to changes in protein expression should also be performed. Additionally, given that *RPE65* was consistently differentially expressed in the brains of monkeys of Chinese and Indian origin, functional studies determining the specific role of this protein in the brain are warranted in order to determine its relationship to the brain concentrations of lutein and zeaxanthin.

The first limitation of this cross-sectional, correlational study is that the results do not provide information on the causal effect of differences in gene expression due to origin on neural lutein and zeaxanthin status, or vice versa. Another limitation is that, unlike the Indian-origin monkeys, Chinese-origin monkeys were imported to the ONPRC as adults. Therefore, we cannot account for earlier long-term epigenetic (e.g., environmental and dietary) factors that may have influenced gene expression in these animals as adults. However, animals lived in the same environment and consumed the same diet for at least 3 years before termination, thus limiting the number of variables for a significant amount of time leading up to blood and tissue collection. A major strength of this study is that these results provide valuable knowledge regarding factors related to variability in carotenoid status, and in particular, lutein and zeaxanthin levels, among genetically diverse rhesus monkeys. This information should be considered when designing and implementing studies on carotenoids in rhesus macaques. Furthermore, numerous studies have linked neural xanthophyll concentrations, particularly lutein, to cognitive performance in humans [6, 8–11]. Therefore, our findings have important implications regarding the influence of genetics on the role of lutein (and zeaxanthin) in cognition in primates. However, future studies must be conducted to evaluate the association between differences in carotenoid-related gene expression profiles and carotenoid-related function in brain tissue in order to determine the functional impact of differences in brain xanthophyll status due to genetics on brain health, function, and overall cognition.

## Additional files

Additional file 1: Table S1. Mean number of reads (± SD) and quality score (PHRED format ± SD) for high and low lutein content in each brain region. **Table S2.** Primer sequences utilized in RT-PCR analysis. (DOCX 12 kb)
Additional file 2: Figure S1. Individual (A) lutein and (B) zeaxanthin concentrations (ng/mg protein) in the prefrontal cortex of Indian- and Chinese-origin rhesus monkeys (*n* = 6). **Figure S2.** Individual (A) lutein and (B) zeaxanthin concentrations (ng/mg protein) in the cerebellum of Indian- and Chinese-origin rhesus monkeys (*n* = 6). **Figure S3.** Individual (A) lutein and (B) zeaxanthin concentrations (ng/mg protein) in striatum of Indian- and Chinese-origin rhesus monkeys (*n* = 6). (DOCX 34 kb)
