# Supplementary material for: Brain xanthophyll content and exploratory gene expression analysis: subspecies differences in rhesus macaque
Source: Genes Nutr. 2017 Mar 8;12:9. doi: 10.1186/s12263-017-0557-3 (PMC5341479; doi:10.1186/s12263-017-0557-3)
Supplement: Additional file 1: Table S1. — Mean number of reads (± SD) and quality score (PHRED format ± SD) for high and low lutein content in each brain region. Table S2. Primer sequences utilized in RT-PCR analysis. (DOCX 12 kb) [file 12263_2017_557_MOESM1_ESM.docx]

**Additional file**

**Table S1.** Mean number of reads (± SD) and quality score (PHRED format ± SD) for high and low lutein content in each brain region

|  | Number of Reads | Mean Quality Score |
| --- | --- | --- |
| Prefrontal Cortex |  |  |
| Indian | 41,761,479 ± 3,293,565 | 34.80 ± 0.00 |
| Chinese | 43,522,584 ± 588,885 | 34.82 ± 0.02 |
| Cerebellum |  |  |
| Indian | 47,902,114 ± 4,730,013 | 34.85 ± 0.01 |
| Chinese | 50,180,395 ± 1,238,260 | 34.84 ± 0.01 |
| Striatum |  |  |
| Indian | 33,834,270 ± 3,448,786 | 34.81 ± 0.02 |
| Chinese | 39,881,422 ± 3,718,910 | 34.81 ± 0.01 |

**Table S2.** Primer Sequences Utilized in RT-PCR analysis

| Gene | Forward | Reverse |
| --- | --- | --- |
| *BCO2* | tggaccttgaggagaccatc | cccagcttatcccatctgaa |
| *RPE65* | gatgcttacgtacgggcaat | ttgtcggtaacctccactcc |
| *ELOVL4* | ttacactgactgccccttcc | gctcacaccatttgctgaaa |
| *LPL* | gctccttcatgtggcgtatt | gtcacacattgctggtggac |
| *FADS1* | gccaaccatgtgttcttcct | gacaagccaaaggctctcac |
| *FADS2* | cctaaaggtgggaggagacc | agagtgaagccagacccaga |
| *CCR1* | tttggtgtcatcaccagcat | gagcctgaaacagcttccac |
| *CD74* | ccaagtatggcaacatgacg | gcatccagctctcaaagacc |
| *CD4* | gaggtggaattgctggtgtt | agctgaggcacagagatggt |
| *LTBR* | tggcattctttctgctcctt | gggtagcaatggctctacca |
| *CXCL12* | agagccaacgtcaagcatct | ctttagcttcgggtcaatgc |
| *SNCA* | tgtgcccagtcatgacattt | ccacaaaatccacagcacac |
| *ACTG1* | gctcctgaaccagtttctgc | agtaacagcccacggtgttc |
