# Supplementary material for: Brain xanthophyll content and exploratory gene expression analysis: subspecies differences in rhesus macaque
Source: Genes Nutr. 2017 Mar 8;12:9. doi: 10.1186/s12263-017-0557-3 (PMC5341479; doi:10.1186/s12263-017-0557-3)
Supplement: Additional file 2: Figure S1. — Individual (A) lutein and (B) zeaxanthin concentrations (ng/mg protein) in the prefrontal cortex of Indian- and Chinese-origin rhesus monkeys (n = 6). Figure S2. Individual (A) lutein and (B) zeaxanthin concentrations (ng/mg protein) in the cerebellum of Indian- and Chinese-origin rhesus monkeys (n = 6). Figure S3. Individual (A) lutein and (B) zeaxanthin concentrations (ng/mg protein) in striatum of Indian- and Chinese-origin rhesus monkeys (n = 6). (DOCX 34 kb) [file 12263_2017_557_MOESM2_ESM.docx]

**Figure S1**. Individual (A) Lutein and (B) Zeaxanthin Concentrations (ng/mg protein) in Prefrontal Cortex of Indian- and Chinese-Origin Rhesus Monkeys (n=6).

**B**

**A**

**Figure S2**. Individual (A) Lutein and (B) Zeaxanthin Concentrations (ng/mg protein) in Cerebellum of Indian- and Chinese-Origin Rhesus Monkeys (n=6).

**B**

**A**

**Figure S3**. Individual (A) Lutein and (B) Zeaxanthin Concentrations (ng/mg protein) in Striatum of Indian- and Chinese-Origin Rhesus Monkeys (n=6).

**B**

**A**
